# Differential introgression among loci across a hybrid zone of the intermediate horseshoe bat (*Rhinolophus affinis*)

**DOI:** 10.1186/1471-2148-14-154

**Published:** 2014-07-09

**Authors:** Xiuguang Mao, Guangjian Zhu, Libiao Zhang, Shuyi Zhang, Stephen J Rossiter

**Affiliations:** 1 Institute of Molecular Ecology and Evolution, East China Normal University, Shanghai 200062, China; 2 School of Biological and Chemical Sciences, Queen Mary University of London, London E1 4NS, UK; 3 Guangdong Entomological Institute, 105 Xingang Xilu, Haizhu Guangzhou, Guangdong Province 510260, China

**Keywords:** Phylogenetic discordance, Adaptive introgression, Speciation, *Rhinolophus*

## Abstract

**Background:**

Hybrid zones formed by the secondary contact of divergent lineages represent natural laboratories for studying the genetic basis of speciation. Here we tested for patterns of differential introgression among three X-linked and 11 autosomal regions to identify candidate loci related to either reproductive isolation or adaptive introgression across a hybrid zone between two Chinese mainland subspecies of the intermediate horseshoe bat *Rhinolophus affinis*: *R. a. himalayanus* and *R. a. macrurus*.

**Results:**

Our results support the previous suggestion that *macrurus* formed when a third subspecies (*R. a. hainanus*) recolonized the mainland from Hainan Island, and that *himalayanus* is the ancestral taxon. However, this overall evolutionary history was not reflected in all loci examined, with considerable locus-wise heterogeneity seen in gene tree topologies, levels of polymorphism, genetic differentiation and rates of introgression. Coalescent simulations suggested levels of lineage mixing seen at some nuclear loci might result from incomplete lineage sorting. Isolation with migration models supported evidence of gene flow across the hybrid zone at one intronic marker of the hearing gene *Prestin*.

**Conclusions:**

We suggest that phylogenetic discordance with respect to the species tree seen here is likely to arise via a combination of incomplete lineage sorting and a low incidence of introgression although we cannot rule out other explanations such as selection and recombination. Two X-linked loci and one autosomal locus were identified as candidate regions related to reproductive isolation across the hybrid zone. Our work highlights the importance of including multiple genomic regions in characterizing patterns of divergence and gene flow across a hybrid zone.

## Background

Our understanding of the genetics of speciation has benefited greatly from studies of hybridizing species, both in the laboratory [1-3] and in the wild [4-6]. In the latter, hybrid zones - geographic regions where genetically distinct populations meet, mate and produce hybrids [7] - have been considered as 'natural laboratories for evolutionary studies' [8]. For evolutionary biologists, hybrid zones offer windows on evolutionary process [9], while they provide the divergent populations themselves with a means to interact with each other. Genes can exchange due to the semi-permeable nature of the genome [10], and this in turn can result in variation in the level of introgression of alleles among different genomic regions [11-14].

Many processes can contribute to patterns of differential introgression, such as natural selection, genetic drift [15], varying recombination rates [16], linkage [4,17], sampling error, or a combination of these processes. Distinguishing these processes from each other is extremely difficult but may be possible with the aid of newly developed analytical approaches (e.g. genomic clines, [18]), and with sampling of increased numbers of loci across the genome that are being surveyed in multiple individuals.

Based on patterns of introgression, several different classes of genomic region in the hybridized genome have been identified. First, there are regions that resist introgression or show reduced levels of introgression, which are often considered to be, or be linked to, putative ‘speciation genes’ [10,11,19]. A number of such genes have been reported in model organisms; for example, in *Drosophila*[3,20] and in the mouse (*Mus*) [2,21]. Second, there are regions that occur in the genomic backgrounds of both hybridizing species, which appear to be selectively neutral and able to flow freely across species boundaries. Finally, there are regions that introgress faster than neutral genes, and these are often considered to be advantageous or beneficial genes. Cases of such adaptive introgression may be promoted by positive selection [22], and have been documented in both plants [23,24] and animals [25-27]. Recent genome scans conducted in mice have revealed that genes involved in olfaction and pheromone responses have undergone adaptive introgression across a hybrid zone, a result that was attributed to the importance of such genes in the survival and reproduction of these organisms [5].

By studying patterns of introgression in hybrid zones, candidate genomic regions that are either speciation genes, or linked to speciation genes, can be identified on the basis of reduced levels of introgression, whereas candidate genomic regions that are either beneficial genes, or linked to beneficial genes, can be detected from increased levels of introgression [4-6,11,15,28]. One advantage of this differential introgression approach is that it can help to identify candidate genomic regions that are likely to be related to reproductive isolation and/or adaptive introgression between hybridizing species even in the absence of any information about the phenotypes that such regions control. This is particularly useful for studies of wild populations from non-model organisms.

**Figure 1:**
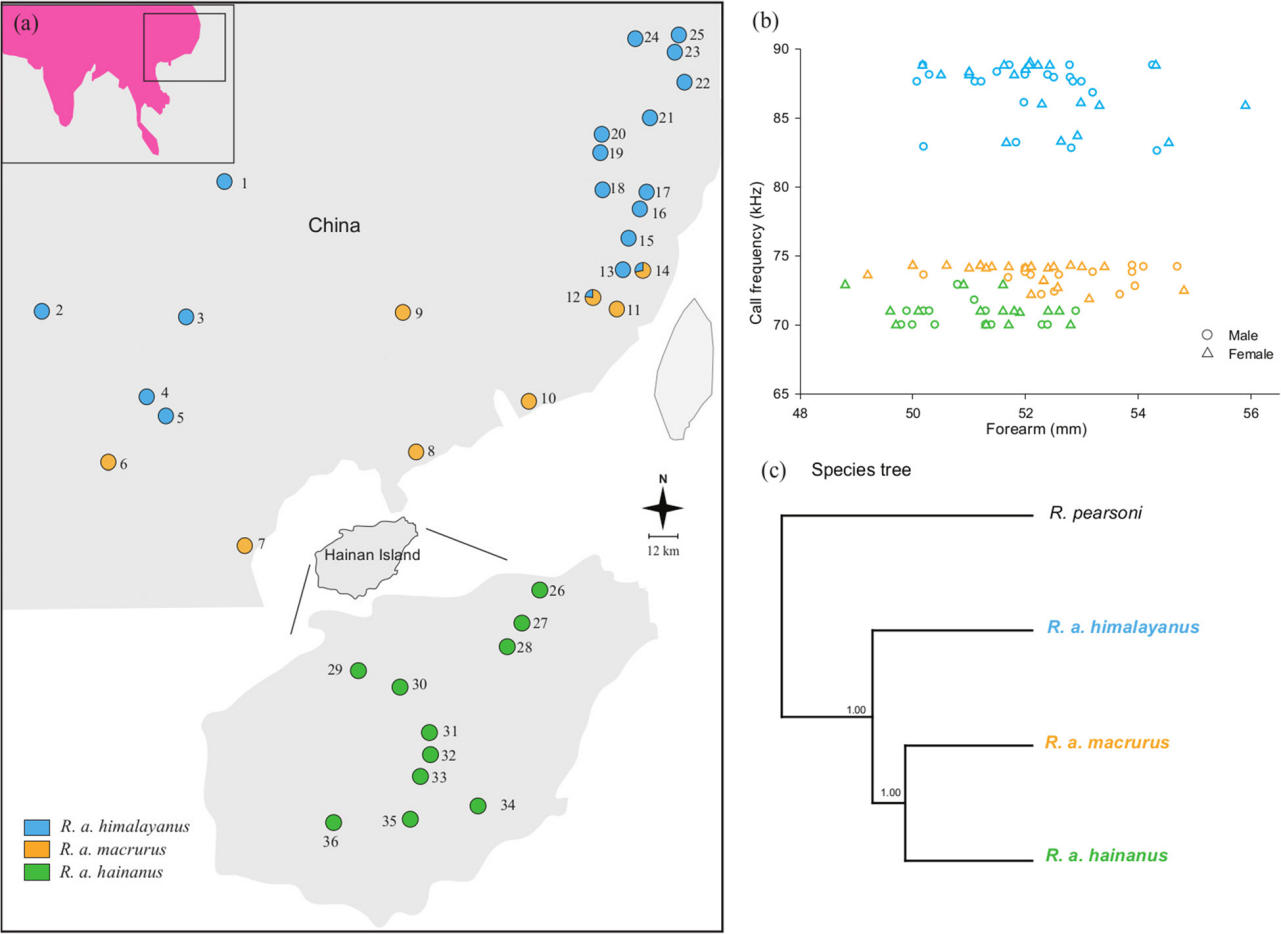
**Sampling, morphological data and species tree. (a)** Map showing the sampling localities of *Rhinolophus affinis* used in this study (modified from Mao *et al.* 2013 [30]). Populations are presented as circles in which individuals are coloured based on the subspecies membership (*R. a. himalayanus*: blue; *R. a. macrurus*: orange; *R. a. hainanus*: green); **(b)** Plot showing echolocation call frequency (kHz) and forearm (mm) data for a subset of bats from the three subspecies (*R. a. himalayanus*, n = 43; *R. a. macrurus*, n = 39; *R. a. hainanus*, n = 32). Downward and upward triangles correspond to male and female bats, respectively. Full details are provided in Additional file 3: Table S3; **(c)** Species tree based on all nuclear markers using BEST.

A hybrid zone between two subspecies of the intermediate horseshoe bat (*Rhinolophus affinis*) provides an opportunity to gain insights into the genomic regions likely to be responsible for reproductive isolation and/or adaptive introgression in these taxa. *R. a. himalayanus* and *R. a. macrurus* both occur on the Chinese mainland (see Figure 1a). Previous phylogeographic studies suggest that the mainland *R. a himalayanus* first colonized Hainan Island to form *R. a. hainanus*, which then underwent a post-glacial recolonization of the mainland to form *R. a. macrurus*[29,30]*.* The two mainland subspecies, *himalayanus* and *macrurus*, now form a hybrid zone in southeastern China. Earlier analyses of the mitochondrial control region, three nuclear genes and 14 microsatellites loci [30] suggested the occurrence of mitochondrial introgression but no nuclear introgression across the hybrid zone, although the absence of detected nuclear introgression may have reflected a lack of data.

To test for differential introgression across the hybrid zone in *R. affinis,* here we expand our sampling of the genome to include loci that might be subject to adaptive introgression. Unlike mice in which olfaction is the dominant sensory modality, horseshoe bats are auditory specialists that use a system of narrowband constant frequency echolocation in which the inner ear is finely tuned to the incoming echoes of the emitted call. These calls have evolved specifically for the detection of flying insects, with the call frequency influencing the prey size and habitat use. For this reason, putative echolocation (or hearing) genes [31] might be subject to adaptive introgression. To test this hypothesis in our study system, we analyzed polymorphism and genetic differentiation in regions from three echolocation genes (i.e. *FoxP2*, *Kcnq4* and *Prestin*). The first of these is implicated in orofacial coordination and vocalisation - both of which are important in call production - and was shown to have undergone divergent selection in echolocating bats [32]. The latter two genes encode proteins involved in hair cell function, and both show extensive sequence convergence between echolocating bats and dolphins [33,34]. In comparison, we included three X-linked and five other autosomal regions that are not expected to have any roles in hearing or vocalisation. The inclusion of X-linked markers in our dataset allowed us to also test the hypothesis that the X chromosome contributes disproportionately to reproductive isolation compared to autosomes [35].

## Methods

### Sampling and echolocation calls recording

Individuals of all three subspecies (*R. a. himalayanus*, *R. a. macrurus* and *R. a. hainanus*) sequenced in this study were sampled previously [29,30] from across the range (Figure 1a). A wing membrane biopsy was taken from each individual following standard non-lethal sampling procedure that was approved by the National Animal Research Authority, East China Normal University (approval ID 20080209). One congeneric species (*R. pearsoni*) was included as an outgroup in the phylogenetic analyses. All DNA samples analyzed were isolated from 3-mm wing membrane biopsies using DNeasy kits (Qiagen).

Echolocation call frequency and body size are useful characters for distinguishing among closely related bat species [36]. To test for divergence in body size and call frequency among the three focal taxa, for a subset of bats we measured the forearm (mm) using dial calipers, and recorded the echolocation call resting frequency (kHz) using the Avisoft UltraSoundGate 116Hnb kit (Avisoft, Berlin, Germany). Prior to taxonomic comparisons, we also tested for sex differences among call frequencies, which have been reported in several horseshoe bat species. Spectrograms were analyzed using Avisoft-SASLab Pro software (Avisoft) and the constant frequency of the second harmonic was extracted.

### Gene selection and sequencing

Sequence data of *Chd1*, *Sws1* and *Usp9x* from individuals of *himalayanus* and *macrurus* were taken from our previous study [30]. To complement these data, here we also sequenced these genes in the *hainanus* island subspecies, and, for all three taxa, we obtained new sequence data from nine additional genes. First, we amplified the complete cytochrome *b* (*Cytb*) gene using the primers and PCR conditions described in [37]. In addition, for each mtDNA clade (see Results), we also amplified introns of two X-chromosomal genes (*Pola1* and *Cx22*) and six autosomal genes (*Tg*, *Thy*, *H2a*, *Kcnq4, FoxP2* and *Prestin*). For *FoxP2* and *Prestin*, we amplified the second and third introns of the former (hereafter called *FoxP2-2* and -*3*) and four introns of the latter (hereafter called *Prestin-4*, -*8*, -*17* and -*18*). Details of all markers examined here are provided in Table 1.

**Table 1:** Annotated function and chromosome information for molecular markers used in this study

**ID**	**Description**	**Chromosome**	**Annotated function**
*Cytb*	Cytochrome *b*	Mitochondrial	Oxidative phosphorylation
*Chd1*	Chromodomain helicase DNA binding protein 1	Autosome	Chromatin binding
*Sws1*	The short-wavelength-sensitive opsin gene	Autosome	Cognition
*H2a*	H2A histone family, member Y	Autosome	Chromatin binding
*Thy*	Thyrotropin	Autosome	Hormone activity
*Tg*	Thyroglobulin	Autosome	Hormone activity
*Prestin*	Solute carrier family 26, member 5	Autosome	Hearing
*FoxP2*	Forkhead box P2	Autosome	Cognition
*Kcnq4*	The voltage-gated potassium channel subfamily KQT member 4	Autosome	Hearing
*Usp9x*	Ubiquitin specific protease 9 X	X chromosome	Alternative splicing
*Pola1*	Polymerase (DNA directed) alpha 1	X chromosome	Chromatin binding
*Cx22*	X-chromosomal open reading frame 22	X chromosome	Alternative splicing

Polymerase chain reactions (PCRs) were performed in 50 μl reaction mixtures containing 10–50 ng DNA, 0.25 mM of each primer and 25 μl Premix Taq polymerase (TaKaRa). The thermocycling profiles for *Tg*, *Thy* and *Usp9x* have been described previously [38-40]. For *H2a*, *Pola1*, *Cx22*, *Prestin-4*, -*8*, -*17* and -*18*, *FoxP2-2* and -*3*, and *Kcnq4*, we used: 95°C for 5 min; 34 cycles of 94°C for 30 s, 54–61°C for 40 s, 72°C for 90 s; 72°C for 10 min. Details of primer information and actual anneal temperatures for each marker have been provided in Additional file 1: Table S1. PCRs were carried out on a PTC-220 thermal cycler (Bio-Rad). DNA sequencing was undertaken with both primers on an ABI PRISM 3700 automated sequencer (Applied Biosystems). For nuclear sequences, when multiple heterozygous sites were present (i.e. more than one double peak was observed in the sequence chromatograms), haplotypes were resolved probabilistically using PHASE 2.1 [41] in the package DnaSP v5 [42]. Sequences were aligned using CLUSTAL_X 1.83 [43] and edited manually. All sequences generated in this study have been deposited in GenBank and accession numbers are given in Additional file 2: Table S2.

### Gene genealogies and species tree

Phylogenetic relationships among the three subspecies were reconstructed based on the *Cytb* sequences using Bayesian inference (BI) implemented in MrBayes 3.1.2 [44]. To test for genealogical discordance across loci, we also performed a BI tree reconstruction for each nuclear region. For *FoxP2*, most individuals were sequenced at both *FoxP2-2* and -*3*, and thus these two segments were concatenated as a single marker (hereafter called *FoxP2*). For *Prestin*, the DNA of some individuals was either too depleted or degraded for use in all segments of *Prestin*; thus each segment was analyzed separately. The best-fit substitution models for each region were determined in MODELTEST 3.0 [45] and are given in Table 2. We performed two simultaneous runs of Metropolis-coupled Markov chain Monte Carlo (MCMC) analysis with the substitution model parameters, each comprising four chains and 10 million generations. Trees and parameters were sampled every 100 generations, and the first 25% of the sampled trees were discarded as burn-in. Because gene genealogies at the population level are often difficult to represent by traditional phylogenetic trees [46], we also examined the relationship among haplotypes by constructing statistical parsimony networks in the package TCS version 1.21 [47].

**Table 2:** Substitution models determined by MODELTEST for each locus

**Locus**	**Substitution models**
*Cytb*	HKY + I + G [I = 0.7084, G = 1.1615]
*Cx22*	HKY
*Usp9x*	HKY
*Pola1*	HKY + I + G [I = 0.8750, G = 0.6921]
*Chd1*	HKY
*H2a*	K81uf + I + G [I = 0.7127, G = 0.7697]
*Sws1*	HKY + G [G = 0.0061]
*Thy*	K81uf + I + G [I = 0.8111, G = 0.8005]
*Tg*	K80 + G [G = 0.0012]
*Prestin-4*	HKY + I + G [I = 0.8206, G = 0.6954]
*Prestin-8*	HKY + I + G [I = 0.8333, G = 0.8597]
*Prestin-17*	HKY + I + G [I = 0.8838, G = 0.7198]
*Prestin-18*	HKY + G [G = 0.0099]
*FoxP2*	K81uf + I + G [I = 0.7737, G = 0.8948]
*Kcnq4*	HKY

Knowledge of the correct species tree is essential for understanding patterns of introgression. However, due to random lineage sorting, individual gene trees often differ from each other and from the species tree [48]. A Bayesian hierarchical model has been proposed to reconstruct the species tree by incorporating information from multiple gene trees [49,50]. We performed a Bayesian hierarchical model using the software BEST 2.0, which implements a MCMC algorithm to estimate the posterior distribution of species trees. For this analysis, all nuclear markers were included and model parameters for each marker were estimated using MODELTEST (Table 2). In total we included 14 individuals for which full sequence datasets were available: six *himalayanus*, four *macrurus*, three *hainanus* and one *R. pearsoni* as an outgroup. We performed two runs of Metropolis-coupled MCMC, each comprising four chains and 10 million generations. Trees and parameters were sampled every 100 generations, and the first 25% of the sampled trees were discarded as a burn-in.

### Analyses of polymorphism, genetic differentiation and neutrality

For each nuclear locus and each subspecies, we calculated the nucleotide diversity (π) using DnaSP, which was also used to count the number of polymorphic sites, fixed and shared mutations between pairs of subspecies. To assess whether genetic differentiation was lower among the two mainland subspecies, as would be expected from introgression in the hybrid zone, we calculated F_ST_[51] at each locus between each pair of taxa in the software ARLEQUIN 3.5 [52]. Significance was assessed based on 1000 permutations.

Deviations from the neutral model for each locus were assessed by estimating Tajima’s D [53] in ARLEQUIN with significance assessed by 1000 permutations. Multilocus neutrality tests were conducted based on the Hudson–Kreitman–Aguade (HKA) method [54] in DnaSP.

### Coalescent simulations of incomplete lineage sorting

Phylogenetic reconstructions of several nuclear loci revealed a paraphyletic relationship between *himalayanus* and [*macrurus* + *hainanus*] (see Results). Because such mixing could theoretically arise from either introgression or incomplete lineage sorting, we undertook coalescent simulations to test for a possible contribution of incomplete sorting to the patterns observed. We followed the method outlined in [55], with modifications described in [56]. For nuclear genes (i.e. *Pola1*, *Sws1*, *Thy*, *Tg*, *H2a*, *Kcnq4*, *Prestin-4*, -*8* and -*18*) that showed mixing between the *himalayanus* and [*macrurus* + *hainanus*] clades, we used Mesquite v2.5 [57] to calculate Slatkin & Maddison's *s* statistic [58], a measure of the degree of lineage sorting assuming no gene flow after splitting. Given two defined lineages, a *s* value of 1 is expected for a locus showing complete reciprocal monophyly, while a value of >1 indicates mixing. Thus for each locus showing an observed *s* value of >1, we used Mesquite to simulate 1,000 coalescent trees under the incomplete lineage sorting model with no migration within the population tree. Then, sequence data were simulated from the coalescent trees using a model of substitution estimated from the empirical data using MODELTEST excluding the outgroup (see Additional file 3: Table S3). The scale factor for each locus was determined by testing several values until mean sequence divergence values within lineages were similar between simulated and empirical data (see Additional file 3: Table S3). Finally, PAUP* [59] was used to reconstruct majority-rule consensus trees from sequence matrices using heuristic parsimony searches. For each locus, we generated a null distribution of *s* values, against which the empirical value was compared and considered as significant if it fell outside of the 95% confidence intervals.

For all coalescent simulations, the divergence time between *himalayanus* and [*macrurus* + *hainanus*] was determined as 550000 years ago (i.e. 275000 generations based on a generation time of two years for horseshoe bats) using a sequence divergence of 0.011 (introgressed haplotypes were excluded in this analysis) and the divergence rate of 0.02/Mya for cytochrome b; effective population size (N_e_) was estimated from Θ-values using MIGRATE-N version 3.3.2 [60]. Each search contained 20 short chains with 5000 sampled genealogies and 2 long chains with 50,000 sampled genealogies. Genealogies were 200 steps apart and the first 10,000 were discarded as burn-in. Two runs were performed to ensure convergence of parameter estimates. The Θ-values were converted to N_e_ using the formula Θ = 4N_e_u (u is mutation rate). The mutation rates of each nuclear locus were determined by calculating the ratio of the average distance between *himalayanus* and [*macrurus* + *hainanus*] for each locus and that for cytb (1 × 10^-8^ per site per year). Simulations were conducted using an N_e_ based on the maximum likelihood estimates of Θ, as well as the 95% upper and lower percentiles.

### Estimates of gene flow

To further test for introgression, we estimated levels of gene flow between *himalayanus* and *macrurus* by implementing isolation-with-migration (IM) models [61] in the program IMa2 [62,63]. We repeated the IM analysis for each of the nine loci (*Pola1*, *Sws1*, *Thy*, *Tg*, *H2a*, *Kcnq4*, *Prestin-4*, -*8* and -*18*) showing evidence of mixing between *himalayanus* and its daughter taxa. The IM model assumes that each locus is free from recombination and selectively neutral. We tested for recombination using the four-gamete test [64] in DnaSP and only the segments without recombination were used. Neutrality tests have been conducted above. Several preliminary runs were performed to establish upper bounds on prior distributions. A final run was conducted with 200 000 genealogies at every 100 steps after a burn-in of 10^6^ steps including twenty Metropolis-coupled chains with a geometric heating scheme: -hfg -hn20 -ha0.96 -hb0.9. A total of 200 000 genealogies were used to perform likelihood ratio tests of the nested models for migration rates [62].

## Results

### Echolocation call frequency and species tree

*Rhinolophus affinis* showed no sex differences in echolocation call frequency, however, significant divergence was observed among the taxa (see Figure 1b and Additional file 4: Table S4). Specifically, the call frequencies of *himalayanus* (87.12 ± SD 2.04 kHz) were significantly higher than those of both *macrurus* (*macrurus*: 73.68 ± 0.74 kHz; t = 39.020, df = 83, P < 0.001) and *hainanus* (70.85 ± 0.94 kHz; t = 42.102, df = 76, P < 0.001). Call frequencies differences between *macrurus* and *hainanus* were slight but also significant (t = 14.189, df = 69, P < 0.001). The sister relationship between *macrurus* and *hainanus*, and the ancestral status of *himalayanus*, were further supported by the species tree estimated by BEST based on all nuclear regions (Figure 1c).

### MtDNA tree and network

**Figure 2:**
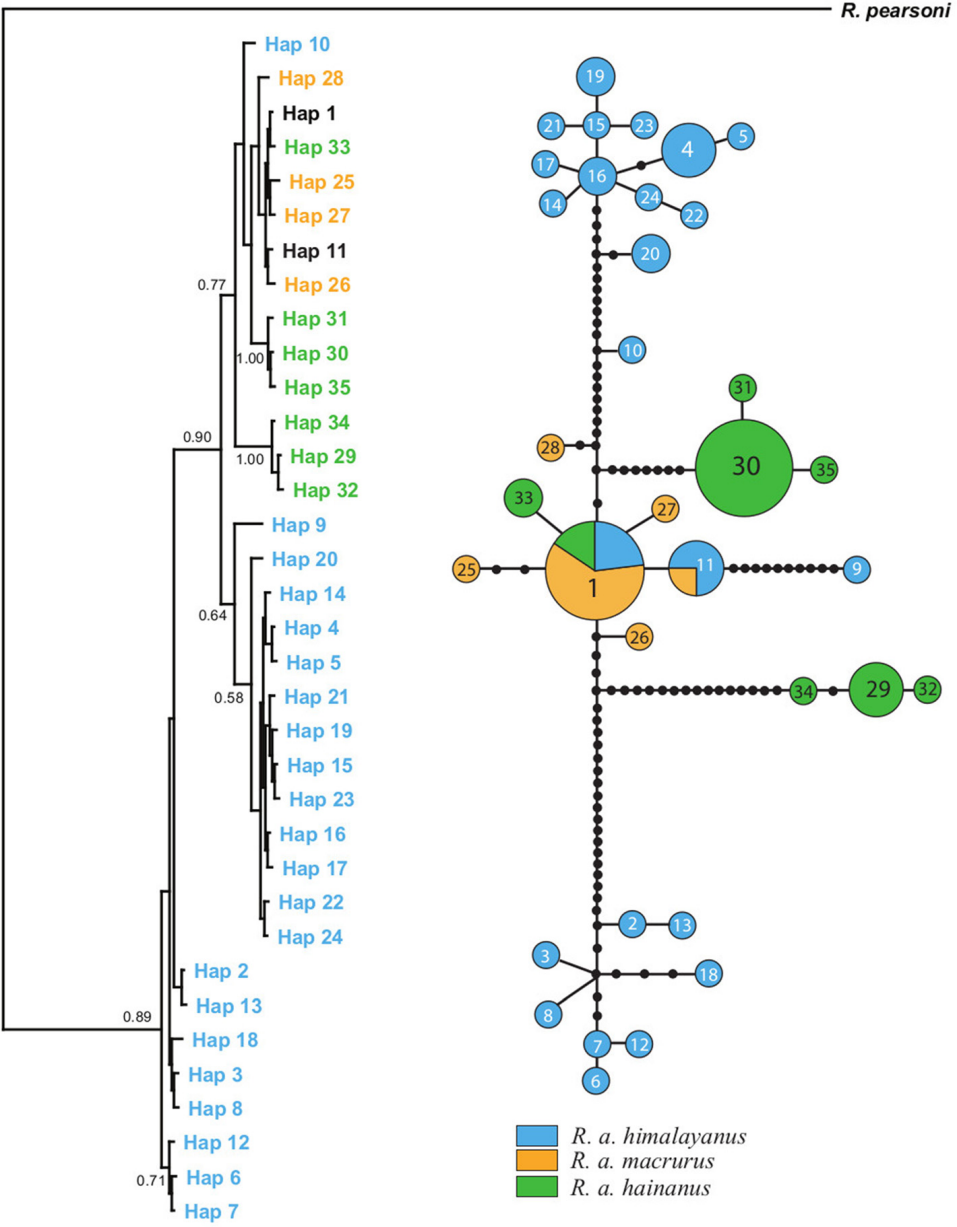
**Bayesian inference (BI) tree and statistical parsimony network based on sequences from Cytochrome b (*Cytb*).** In trees, node support is indicated with Bayesian posterior probabilities and only posterior probabilities over 0.5 are shown. Shared haplotypes between pairs of subspecies are shown in black. In networks, each circle represents a single haplotype and the area of circle size is scaled by haplotype frequency. The filled black circles represent missing or unsampled haplotypes.

The final alignment (1023 bp) of 73 *Cytb* sequences identified 35 haplotypes among the three taxa. No premature stop codons were observed at this gene, suggesting that it is functional. A Bayesian tree and network based on all *Cytb* haplotypes gave a similar pattern to an earlier tree based on the Control Region (CR) [29]. Specifically, haplotypes of *macrurus* were nested within a predominantly *hainanus* clade (Figure 2) supporting the previous suggestion that *macrurus* originated from the recolonisation of *hainanus* onto the mainland. In this tree, most *himalayanus* haplotypes appeared to be ancestral with respect to these other taxa, and formed two clusters with one corresponding to a specific geographical region (see [30]). However, several individuals’ *Cytb* haplotypes - hap1, hap10 and hap11 - were classified with the [*macrurus* + *hainanus*] clade, supporting mtDNA introgression between the two mainland subspecies (see also [29,30]).

### Discordance in genealogical topologies among nuclear loci

**Figure 3:**
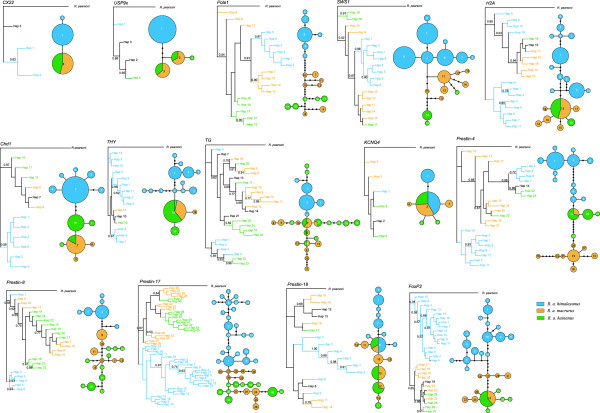
**Bayesian inference (BI) trees and statistical parsimony networks based on sequences from each nuclear region.** In trees, node support is indicated with Bayesian posterior probabilities and only posterior probabilities over 0.5 are shown. Shared haplotypes between pairs of subspecies are shown in black. In networks, each circle represents a single haplotype and the area of circle size is scaled by haplotype frequency. The filled black circles represent missing or unsampled haplotypes.

To test for phylogenetic discordance among genomic regions, phylogenetic trees and networks were reconstructed for all nuclear regions examined (Figure 3). Five regions (i.e. *Cx22*, *Usp9x*, *Chd1*, *Prestin-17*, and *Foxp2*) recovered phylogenetic relationships among the three taxa that were similar to those seen in the mtDNA and species trees, with *macrurus* and *hainanus* haplotypes mixed and highly divergent from *himalayanus* haplotypes. However, eight regions (i.e. *Pola1*, *Sws1*, *Thy*, *Tg*, *H2a*, *Prestin-4*, *Prestin-8*, and *Prestin-18*) produced genealogies in which some haplotypes of *himalayanus* were classified with [*macrurus* + *hainanus*]. In particular, in *Pola1* and *Sws1*, all *himalayanus* haplotypes clustered with some of the *macrurus* haplotypes with high posterior probabilities (Figure 3), indicating possible gene flow between these two mainland subspecies. One gene (*Kcnq4*) showed mixed haplotypes among all three subspecies (Figure 3), possibly due to its low mutation rate.

### Polymorphisms and genetic differentiation, and neutral test

The proposed recent origin of *macrurus* by the recolonization of *hainanus* onto the mainland would be expected to lead to lower levels of nucleotide diversity in *macrurus* than in either *hainanus* or *himalayanus*. However, locus-wise nucleotide diversity was not seen to differ significantly between *macrurus* and *hainanus* (t = -1.4055, df = 13, P = 0.1833, paired t-test) with 50% of markers (*Pola1*, *H2a*, *Tg*, *Prestin-4*, *Prestin-18*, *FoxP2* and *Kcnq4*; see Table 3) showing higher pairwise nucleotide diversity values in the former taxon.

**Table 3:** **Estimates of polymorphism (nucleotide diversity (π) and Tajima's D) within each subspecies and genetic differentiation (F**_**ST**_**) between pairs of subspecies for each nuclear region**

	**N**			**π**			**Tajima's D**			**F**_**ST**_		
**Locus**	***him***	***mac***	***hai***	***him***	***mac***	***hai***	***him***	***mac***	***hai***	***him-mac***	***hai-mac***	***him-hai***
*Cx22*	28	13	12	0.017	0	0	-1.156	0	0	0.95	0	0.95
*Usp9x*	42	12	9	0	0.079	0.116	0	1.381	0.196	0.94	-0.06	0.94
*Pola1*	20	11	8	0.287	1.187	0.404	-0.509	0.823	1.293	0.67	0.34	0.81
*Chd1*	42	15	17	0.067	0.036	0.089	-1.726*	-1.503	0.103	0.91	0.61	0.86
*H2a*	25	15	10	1.019	0.289	0.097	1.064	-0.931	0.426	0.54	0.09	0.54
*Sws1*	48	12	7	0.353	0.267	0.416	0.944	-1.288	-1.576*	0.54	0.62	0.77
*Thy*	17	11	17	0.759	0.041	0.073	0.658	-1.129	0.292	0.47	0.08	0.53
*Tg*	20	10	10	0.213	0.895	0.802	-1.156	0.170	-0.108	0.56	0.14	0.50
*Prestin-4*	23	12	9	0.518	0.579	0.284	-0.133	-1.062	-1.446	0.62	0.64	0.73
*Prestin-8*	14	9	8	0.193	0.544	0.547	-1.246	1.179	0.637	0.59	0.23	0.71
*Prestin*-17	14	9	10	0.646	0.439	0.567	0.136	0.087	1.876	0.36	0.26	0.38
*Prestin-18*	13	11	9	0.784	0.435	0.330	1.103	0.185	1.456	0.43	0.06	0.46
*FoxP2*	23	12	9	0.315	0.204	0.054	0.486	0.327	-1.385	0.65	0.17	0.73
*Kcnq4*	16	11	8	0.164	0.081	0.064	1.999	-0.532	-1.401	0.31	0.06	0.28

N is the number of individuals analyzed for each nuclear region within each subspecies. *him* represents *himalayanus*; *mac* represents *macrurus*; *hai* represents *hainanus*. *P < 0.05.

Under a scenario of lineage sorting with no gene flow during the divergence of the three taxa, the most recently diverged *hainanus* and *macrurus* should share more ancestral polymorphisms. Consistent with this expectation, no loci showed fixed differences between *hainanus* and *macrurus* compared with nine loci between *himalayanus* and *macrurus*, and ten loci between *himalayanus* and *hainanus* (Table 4). Likewise, levels of genetic differentiation were significantly lower between *hainanus* and *macrurus* (average F_ST_ = 0.23) than between *himalayanus* and *macrurus* (average F_ST_ = 0.62, t = -5.063, df = 28, P < 0.0001), and between *himalayanus* and *hainanus* (average F_ST_ = 0.66, t = -5.561, df = 28, P < 0.0001). On the other hand, six loci - *Pola1*, *Sws1*, *Thy*, *Foxp2*, *Prestin-4*, and *Prestin-8* - showed lower differentiation between *himalayanus* and *macrurus* than between *himalayanus* and *hainanus* (t = -3.531, df = 10, P < 0.05), again suggesting possible introgression between *himalayanus* and *macrurus*. The multilocus HKA test suggested no departure from neutrality for any loci, although the standard neutrality test based on Tajima's D was significant for *Chd1* in *himalayanus* (Table 3).

**Table 4:** Polymorphism and fixed difference between pairs of subspecies

	**Polymorphic sites**			**Fixed differences**			**Shared mutations**		
**Locus**	***him-mac***	***hai-mac***	***him-hai***	***him-mac***	***hai-mac***	***him-hai***	***him-mac***	***hai-mac***	***him-hai***
*Cx22*	2	0	2	1	0	0	0	0	0
*Usp9x*	2	2	3	1	0	1	0	1	0
*Pola1*	17	16	12	1	0	4	3	2	1
*Chd1*	12	4	11	4	0	3	0	0	0
*H2a*	20	7	16	1	0	1	1	0	0
*Sws1*	16	13	17	1	0	2	0	3	0
*Thy*	11	2	12	0	0	0	1	0	0
*Tg*	19	21	17	0	0	1	0	5	3
*Prestin-4*	22	17	18	0	0	2	6	7	3
*Prestin-8*	16	14	17	1	0	2	1	5	2
*Prestin*-17	27	14	25	0	0	0	4	7	4
*Prestin-18*	14	6	11	0	0	0	1	3	1
*FoxP2*	30	13	29	4	0	8	0	0	0
*Kcnq4*	4	4	4	0	0	0	0	0	0

*him* represents *himalayanus*; *mac* represents *macrurus*; *hai* represents *hainanus*.

### Coalescent simulations of incomplete lineage sorting

**Figure 4:**
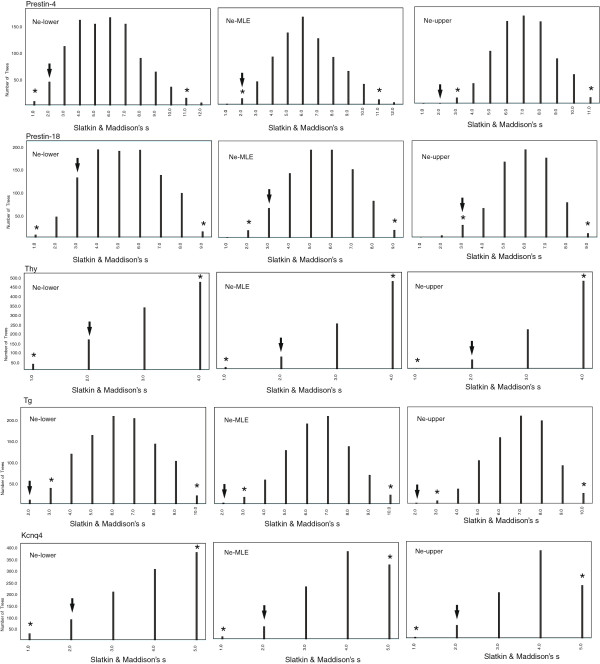
**Distributions of Slatkin & Maddison's*s *for 1,000 simulated trees within population trees from five nuclear loci at the lower, upper and maximum likelihood estimates (MLE) of the effective population size estimated by MIGRATE-N.** The arrow highlights the *s*-value from the empirical data. The 95 % confidence intervals generated from simulations were shown with asterisks.

Four (*Sws1*, *Pola1*, *H2a* and *Prestin-8*) of the nine loci that showed mixing between the *himalayanus* and [*macrurus* + *hainanus*] clades were characterized by *s*-statistic values of 1, indicating complete reciprocal monophyly at these loci. The remaining five loci were characterized by *s*-statistic values of >1 (the *s* value for *Prestin-4*, *Thy*, *Tg* and *Kcnq4* was 2 and for *Prestin-18* was 3), indicating a lack of reciprocal monophyly between *himalayanus* and [*macrurus* + *hainanus*]. The scenario of incomplete lineage sorting with no migration could not be rejected in four of loci (*Prestin-4*, *Thy*, *Kcnq4* and *Prestin-18*) with the values of *s* falling within the 95% confidence intervals generated from simulation tests (Figure 4). In contrast, the *s*-value for *Tg* was significantly small, falling in the lower 5% tail of values, suggesting that it probably did not reflect incomplete lineage sorting.

### Estimates of gene flow

We compared the fit of nested models with zero gene flow for each of the nine loci using IMa2. The likelihood ratio test rejected the model with zero gene flow only at *Prestin-4* from *macrurus* to *himalayanus* (P = 0.043) (see details in Additional file 5: Table S5).

## Discussion

In this study the species tree estimated using 14 loci under a Bayesian hierarchical model and the phylogeny constructed from mitochondrial DNA, together with call frequency variation, supports our earlier proposal that *himalayanus* colonized Hainan Island to form *hainanus*, which later recolonized the mainland forming *macrurus*[29,30]. The recent origin of *macrurus* from *hainanus* was also supported by its comparatively lower average genetic differentiation (Table 3) and almost no fixed differences seen in nuclear markers here (Table 4).

Despite these patterns, the same evolutionary history was not suggested by all 14 nuclear loci. Indeed, nine loci showed evidence of mixing between *himalayanus* and its daughter taxa based on their genealogical topologies. However, four of those loci (*Sws1*, *H2a*, *Pola1* and *Prestin-8*) gave the *s*-statistic value of 1, indicating complete reciprocal monophyly in these loci. This was confirmed by the IMa2 analysis that indicated that the model of zero gene flow could not be rejected in these loci. In the remaining five loci (*Prestin-4*, *Thy*, *Tg*, *Kcnq4* and *Prestin-18*) with the *s*-statistic values > 1, the absence of reciprocal monophyly might have plausibly arisen from incomplete lineage sorting although low levels of introgression might also contribute to this pattern, e.g. at *Prestin-4*. Additionally, differences in locus-specific recombination rates can also lead to variation in levels of genetic differentiation and gene flow [16,65,66]. Our findings add to several recent studies of closely related taxa that have also shown that genealogical patterns and/or levels of genetic differentiation and gene flow can vary considerably across different genomic regions [67-69].

Of the three X-linked markers studied, two (*Cx22* and *Usp9x*) showed extremely low levels of nucleotide diversity (π < 0.12%) within all three subspecies, alongside high genetic differentiation (F_ST_ > 0.94) between *himalayanus* and the other taxa, in accordance with a reciprocal monophyly for *himalayanus* and [*macrurus* + *hainanus*]. Such signatures could arise from selective sweeps operating to reduce genetic diversity within each taxon while fixing divergent alleles in different taxa [70]. These patterns are consistent with the idea that the X-chromosome plays a greater role than autosomes in reproductive isolation [4,5,71]. In contrast, however, the third X-linked locus (*Pola1*) showed highest nucleotide diversity in *macrurus* (the youngest taxon) than any of the other markers. Moreover, this locus also showed both a closer phylogenetic relationship and low estimated genetic differentiation between *himalayanus* and *macrurus* than between *macrurus* and *hainanus*, both of which likely resulted from either incomplete lineage sorting or introgression between *himalayanus* and *macrurus*. However, both of these two scenarios were not supported by the estimated *s*-value of 1, or by the nested migration models performed in IMa2. Thus, other processes such as recombination might also contribute to the patterns observed in *Pola1* (see also [68]).

The IMa2 analysis indicated that the model with zero gene flow could be rejected for *Prestin-4*, supporting our original hypothesis that candidate echolocation genes may be subject to adaptive introgression (see Background). On the other hand, the other two candidate echolocation genes (*FoxP2* and *Kcnq4*) examined showed no evidence of introgression across the hybrid zone and, therefore, we cannot confidently associate the introgression of *Prestin* with its role in echolocation. Additional candidate hearing and/or echolocation genes must be studied for a more thorough assessment of whether there are differences in levels of introgression between hearing genes and other genes in these bats. Indeed, tests for selection for this region of *Prestin* were not significant, suggesting that the possible occurrence of introgression in *Prestin-4* may in fact be due to neutral gene flow although we cannot reject the alternative possibility of linkage to beneficial genes [27,72,73]. It was noteworthy that the scenario of incomplete lineage sorting could not be ruled out completely, and it is possible that the parapyhly pattern at this locus might have been shaped by a combination of both introgression and incomplete lineage sorting. Finally, we were unable to assess the effects of recombination rate on patterns of introgression [16,68] due to lack of detailed knowledge of the genomic locations of the genes examined, such as whether they occur within rearranged or colinear regions of the genome. Beside the possible occurrence of gene flow across the hybrid zone, data from levels of polymorphism, genetic differentiation and genealogical topology suggested that locus *Chd1* may represent a candidate region associated with reproductive isolation, either directly or via linkage to another locus.

Detected possible gene flow between *himalayanus* and *macrurus* at *Prestin* conflicts with our earlier findings for which no nuclear gene flow was inferred on the basis of reciprocal monophyletic tree topologies despite extensive mtDNA introgression [29,30]. Interestingly three nuclear genes (*Chd1*, *Sws1* and *Usp9x*) did not show evidence of gene flow in this study. Given these differences among loci, our results highlight the importance of including multiple genomic regions in order to characterize patterns of introgression across a hybrid zone. This differential introgression pattern across loci observed here has been widely documented in other taxa including both plants (e.g. sunflowers [24] and trees [74]) and animals (e.g. tiger salamanders [25], mice [26], butterflies [27], and crickets [75]. It is pertinent that a recent study has shown that this heterogeneity among loci can affect the inference of the history of speciation if it is not taken into account [76].

## Conclusions

By comparing patterns of divergence and gene flow among loci we identified several regions with putative roles in either reproductive isolation (e.g. *Cx22*, *Usp9x* and *Chd1*) or adaptive introgression (e.g. *Prestin*) in the hybrid zone between *himalayanus* and *macrurus*. However, incomplete lineage sorting, as an alternative scenario for introgression, could not be ruled out completely in this study. Nonetheless a fuller understanding of the factors driving the process of differential introgression will benefit from the number of markers used and knowledge of genomic locations or functional/linkage relationships among those markers. Regardless, our findings add to mounting evidence that caution must be exercised when drawing conclusions about the occurrence of nuclear introgression on the basis of a small number of loci.

## Competing interests

The authors declare that they have no competing interests.

## Authors’ contributions

XM, SZ, SR conceived the project. XM conducted the experiments and the data analysis and wrote the draft. GZ and LZ collected samples. SR edited the draft. SZ provided funding support. All authors read and approved the final manuscript.

## Supplementary Material

Additional file 3: Table S3 Detailed information of the forearm and echolocation call frequency for each individual used in this study. Click here for file

Additional file 1: Table S1 Detailed information on the sequences of the primers, anneal temperature in PCR and references for each marker. Click here for file

Additional file 2: Table S2 List of the GenBank accession numbers for all the sequences included in this study. Click here for file

Additional file 4: Table S4 Parameters used for nuclear markers in coalescent simulations. Click here for file

Additional file 5: Table S5 Tests of nested models for migration rates between *himalayanus* and *macrurus* based on the full data set. Click here for file
