# Supplementary material for: Differential introgression among loci across a hybrid zone of the intermediate horseshoe bat (Rhinolophus affinis)
Source: BMC Evol Biol. 2014 Jul 9;14:154. doi: 10.1186/1471-2148-14-154 (PMC4105523; doi:10.1186/1471-2148-14-154)
Supplement: Additional file 3: Table S3 — Detailed information of the forearm and echolocation call frequency for each individual used in this study. [file 1471-2148-14-154-S3.doc]

Additional files:

Additional file 3: Parameters used for each locus in coalescent simulations.

| Locus | Length | Mode of sequence evolution | N_e-lower_ | N_e-MLE_ | N_e-upper_ | Mutation rate | Scale factor |
| --- | --- | --- | --- | --- | --- | --- | --- |
| *Prestin-4* | 568 | I=0.8546; G=0.4042;Ti/tv=5.2156;  freqA=0.2428, freqC=0.1933, freqG=0.2426, freqT=0.3213 | 944 444 | 1 522 222 | 2 438 889 | 4.5x10^-9^ | 4 x10^-9^ |
| *Prestin-18* | 411 | I=0.9289; Ti/tv=1.7634;  freqA=0.3127, freqC=0.1771, freqG=0.1634, freqT=0.3467 | 1 194 444 | 2 138 889 | 4 009 259 | 2.7x10^-9^ | 5 x10^-9^ |
| *Thy* | 444 | I=0.9403; G=0.7962;  freqA=0.2539, freqC=0.2022, freqG=0.2041, freqT=0.3401 | 1 074 074 | 1 944 444 | 3 574 074 | 2.7x10^-9^ | 6 x10^-9^ |
| *Tg* | 466 | I=0.9107; Ti/tv=3.4799; | 1 840 278 | 2 861 111 | 4 493 056 | 3.6x10^-9^ | 2.5 x10^-9^ |
| *Kcnq4* | 629 | freqA=0.1836, freqC=0.4398, freqG=0.2105, freqT=0.1661 | 1 166 667 | 2 388 889 | 5 740 741 | 2.7x10^-9^ | 1 x10^-9^ |
