# Supplementary material for: Differential introgression among loci across a hybrid zone of the intermediate horseshoe bat (Rhinolophus affinis)
Source: BMC Evol Biol. 2014 Jul 9;14:154. doi: 10.1186/1471-2148-14-154 (PMC4105523; doi:10.1186/1471-2148-14-154)
Supplement: Additional file 1: Table S1 — Detailed information on the sequences of the primers, anneal temperature in PCR and references for each marker. [file 1471-2148-14-154-S1.doc]

Additional files:

Additional file 1: Detailed information on the sequences of the primers, anneal

temperature in PCR and references for each marker.

| ID | Length (bp) | Primers (5′->3′) | Anneal  temperature | References |
| --- | --- | --- | --- | --- |
| *Cytb* | 1023 | F: TAGAATATCAGCTTTGGGTG  R: AAATCACCGTTGTACTTCAAC | 55 °C | Mao *et al.* 2010a |
| *Chd1* | 656 | F: GATAARTCAGARACAGACCTTAGACG  R: TTTGGCATTCACCTGYACTCC | 55 °C | Lim *et al*. 2008 |
| *Sws1* | 645 | F: CACAGGCTATGGTGCTGACTT  R: GCCCGTGGGGATGGCTATTGA | 61 °C | Mao *et al.* 2010a |
| *H2a* | 398 | F: GGACAACAAGAAGGGACG  R: TGGGTGGATGTTTGGTAA | 61 °C | This study |
| *Thy* | 444 | F: GGGTATGTAGTTCATCTTACTTC  R: GGCATCCTGGTATTTCTACAGTCTTG | 55 °C | Eick et al. 2005 |
| *Tg* | 466 | F: GAGCCCAAGCAATGTAAGTCT  R: ATACCACTCGAAGGCCTGCTC | 55 °C | Matthee *et al*. 2001 |
| *Prestin-4* | 568 | F: GAGGAGTAAATGCGACCAA  R: ATCCCACTGTACCGCTTTG | 56 °C | This study |
| *Prestin-8* | 669 | F: GCTCCTACCTCCAGCCAATC  R: ATTGCGAAAGTCTGGAAGAGCGAGC | 58 °C | This study |
| *Prestin-17* | 772 | F: GATGTCGGTATTTATGTGTATTTAG  R: ACAGCAGGTCCAGTAAGG | 56 °C | This study |
| *Prestin-18* | 411 | F: GTAAAGGAATATGGAGATGTCGG  R: CTTGCTATGCGTCATTCACCCTC | 61 °C | This study |
| *FoxP2-2* | 600 | F: AGTTTAGGCTATGGAGCA  R: CTGTTCCCGTTACTGTCG | 56 °C | This study |
| *FoxP2-3* | 874 | F: GCTTACCTCAAACCCCTACCA  R: CCTGAAGTAAGCAAATGTCCG | 61 °C | This study |
| *Kcnq4* | 646 | F: GCGTGGTCAAGGTGGAGA  R: GCAGGCAGCGTGAATAGAA | 61 °C | This study |
| *Usp9x* | 674 | F:GGCAGACAGGTTGATGACTTGGA  R:AGGTCTGCAACTTGCCAAAGGAA | 56 °C | Lim *et al*. 2008 |
| *Pola1* | 549 | F:GAAACTGGTAGAGCGGAGAA  R: ACCTCCCTTCCTTTGTATG | 58 °C | This study |
| *Cx22* | 446 | F: CAAAATGATTCAGTTGGAGA  R: CCCTTCATTAGGAACACAGGAGATA | 54 °C | This study |
