# Supplementary material for: Differential introgression among loci across a hybrid zone of the intermediate horseshoe bat (Rhinolophus affinis)
Source: BMC Evol Biol. 2014 Jul 9;14:154. doi: 10.1186/1471-2148-14-154 (PMC4105523; doi:10.1186/1471-2148-14-154)
Supplement: Additional file 2: Table S2 — List of the GenBank accession numbers for all the sequences included in this study. [file 1471-2148-14-154-S2.doc]

Additional files:

Additional file 2: List of the GenBank accession numbers for all sequences used in this study. *him* represents *himalayanus*; *mac* represents *macrurus*; *hai* represents *hainanus*. Sequences in bold were cited from the previous study[30].

| ID | Taxon | Locality | Locus |  |  |  |  |  |  |  |  |  |  |  |  |  |  |  |
| --- | --- | --- | --- | --- | --- | --- | --- | --- | --- | --- | --- | --- | --- | --- | --- | --- | --- | --- |
|  |  |  | Cytb | Thy | Tg | H2a | Kcnq4 | Pola1 | FoxP2-2 | FoxP2-3 | Cx22 | Prestin-4 | Prestin-8 | Prestin-17 | Prestin-18 | Sws1 | Chd1 | Usp9x |
| QX01 | *him* | 14 |  | KJ623076-77 | KJ623145 | KJ622670 | KJ622726 | KJ622774 | KJ622533 | KJ622598 | KJ622397 | KJ622832-33 | KJ622887 | KJ622929-30 | KJ623011-12 |  | **JN082019** |  |
| FWY010 | *him* | 16 | KJ622453 |  | KJ623138 | KJ622659-60 | KJ622725 | KJ622781 | KJ622546-47 | KJ622611-12 | KJ622410 | KJ622815 | KJ622882-83 |  |  | **JN082079** | **JN082025** | **JN082159** |
| FGS002 | *him* | 16 | KJ622449 |  | KJ623143-44 | KJ622665-66 |  | KJ622775 | KJ622552-53 | KJ622617-18 | KJ622416 |  | KJ622893 | KJ622944-45 |  |  | **JN082021** | **JN082158** |
| YH04 | *him* | 15 | KJ622454 | KJ623078 |  |  |  |  |  |  |  |  |  |  |  | **JN082080-81** | **JN082020** | **JN082157** |
| ST05 | *him* | 23 | KJ622444 | KJ623091 | KJ623127 | KJ622675-76 | KJ622735-36 | KJ622788 | KJ622534-35 | KJ622599-600 | KJ622411 | KJ622843 | KJ622880 | KJ622935 | KJ623021 | **JN082117** | **JN082018** | **JN082125** |
| HS10 | *him* | 22 |  | KJ623085-86 | KJ623149 | KJ622646 |  |  | KJ622529-30 | KJ622594-95 | KJ622395 | KJ622818-19 | KJ622885-86 | KJ622957-58 |  |  |  |  |
| XT01 | *him* | 1 | KJ622468 | KJ623092-93 | KJ623148 | KJ622681 |  | KJ622773 | KJ622538-39 | KJ622603-04 | KJ622409 | KJ622817 | KJ622895-96 |  |  | **JN082113** | **JN082058** | **JN082150** |
| YL24 | *him* | 24 |  | KJ623089-90 |  | KJ622651 | KJ622727 | KJ622785 | KJ622527-28 | KJ622591-93 | KJ622399 | KJ622820-21 |  | KJ622942-43 | KJ622999-3000 | **JN082117** |  | **JN082129** |
| BSC01 | *him* | 21 | KJ622455 |  |  |  |  |  |  |  |  |  |  |  |  | **JN082088** | **JN082035** | **JN082131** |
| FSC01 | *him* | 18 |  | KJ623066-67 |  | KJ622671-72 | KJ622743-44 | KJ622786 | KJ622540-41 | KJ622605-06 | KJ622417 | KJ622809-10 |  |  |  | **JN082089** | **JN082036** |  |
| FSC02 | *him* | 18 |  | KJ623068 |  | KJ622673-74 |  |  | KJ622532 | KJ622597 | KJ622396 | KJ622811-12 | KJ622888-89 | KJ622950-51 |  |  |  |  |
| FSC03 | *him* | 18 |  | KJ623069-70 |  |  |  |  |  |  |  |  |  |  |  |  |  |  |
| FSC06 | *him* | 18 |  | KJ623071 |  |  |  |  |  |  |  |  |  |  |  | **JN082090** | **JN082037** | **JN082132** |
| FSC12 | *him* | 18 |  |  |  |  |  |  |  |  |  |  |  |  |  | **JN082091** | **JN082038** | **JN082133** |
| LC02 | *him* | 5 | KJ622464 |  |  | KJ622687-88 |  | KJ622787 |  |  |  | KJ622836 | KJ622894 | KJ622933-34 |  | **JN082112** | **JN082054** |  |
| LC06 | *him* | 5 |  |  |  |  |  |  |  |  |  |  |  |  |  |  | **JN082055** |  |
| LC07 | *him* | 5 |  |  |  |  |  |  |  |  |  |  |  |  |  |  | **JN082056** |  |
| YJ04 | *him* | 4 |  | KJ623079-80 | KJ623147 | KJ622683-84 | KJ622731 | KJ622784 | KJ622536 | KJ622601 | KJ622413 | KJ622834-35 | KJ622890 | KJ622940-41 | KJ623003-04 |  |  |  |
| YJ03 | *him* | 4 | KJ622469 |  |  |  |  |  |  |  |  |  |  |  |  | **JN082116** |  | **JN082153** |
| YJ05 | *him* | 4 | KJ622470 |  |  |  |  |  |  |  |  |  |  |  |  |  |  |  |
| YJ06 | *him* | 4 | KJ622471 |  |  |  |  |  |  |  |  |  |  |  |  |  |  |  |
| YJ14 | *him* | 4 |  |  |  |  |  |  |  |  |  |  |  |  |  |  | **JN082061** |  |
| YJ15 | *him* | 4 |  |  |  |  |  |  |  |  |  |  |  |  |  |  | **JN082062** |  |
| TC05 | *him* | 2 | KJ622465 |  |  |  |  |  |  |  |  |  |  |  |  |  |  |  |
| TC06 | *him* | 2 | KJ622466 |  |  |  |  |  |  |  |  |  |  |  |  |  |  |  |
| TC07 | *him* | 2 | KJ622467 |  |  |  |  |  |  |  |  |  |  |  |  |  |  |  |
| TC14 | *him* | 2 |  | KJ623081-82 | KJ623139 | KJ622682 | KJ622732 | KJ622782 | KJ622542-43 | KJ622607-08 | KJ622418 | KJ622842 |  |  | KJ623017-18 | **JN082114-15** | **JN082059** | **JN082154** |
| TC15 | *him* | 2 |  |  |  |  |  |  |  |  |  |  |  |  |  |  | **JN082060** |  |
| XGD01 | *him* | 3 | KJ622472 | KJ623094 |  | KJ622677-78 | KJ622729 | KJ622789 |  |  | KJ622407 | KJ622837-38 | KJ622881 | KJ622955-56 |  |  |  | **JN082152** |
| XGD05 | *him* | 3 | KJ622473 |  |  |  |  |  |  |  |  |  |  |  |  |  | **JN082057** |  |
| XGD06 | *him* | 3 |  |  |  |  |  |  |  |  |  |  |  |  |  | **JN082110** |  |  |
| XGD09 | *him* | 3 | KJ622474 |  |  |  |  |  |  |  |  |  |  |  |  |  |  |  |
| XGD10 | *him* | 3 | KJ622475 |  | KJ623150 | KJ622679-80 | KJ622733-34 | KJ622778 | KJ622525-26 | KJ622590-91 |  | KJ622839 | KJ622897 | KJ622952-53 | KJ623022 |  |  |  |
| XGD14 | *him* | 3 | KJ622476 |  |  |  |  |  |  |  |  |  |  |  |  |  |  |  |
| XGD15 | *him* | 3 | KJ622477 |  |  |  |  |  |  |  |  |  |  |  |  |  |  |  |
| XGD020 | *him* | 3 |  | KJ623087-88 |  |  |  |  |  |  |  |  |  |  |  |  |  |  |
| YN01 | *him* | 3 |  |  |  |  | KJ622730 |  |  |  |  |  |  |  | KJ623001-02 |  |  |  |
| YN03 | *him* | 3 |  | KJ623083-84 | KJ623146 | KJ622685-86 |  | KJ622779 |  |  | KJ622415 | KJ622840-41 | KJ622884 | KJ622931-32 |  | JN082111 |  | **JN082151** |
| FJK005 | *him* | 12 | KJ622448 |  | KJ623141 | KJ622668-69 |  |  | KJ622531 | KJ622596 | KJ622412 |  |  | KJ622946-47 |  | **JN082077-78** | **JN082022-23** | **JN082155** |
| FJK022 | *him* | 12 | KJ622447 |  | KJ623137 | KJ622667 | KJ622745-46 | KJ622777 | KJ622523 | KJ622588 | KJ622414 | KJ622813-14 |  | KJ622948-49 | KJ623015-16 | **JN082076** | **JN082024** | **JN082156** |
| WYS084 | *him* | 17 | KJ622450 |  | KJ623140 | KJ622661-62 | KJ622739-40 | KJ622776 |  | KJ622586-87 | KJ622405 |  |  | KJ622961-62 |  | **JN082082** | **JN082026** | **JN082160** |
| WYS085 | *him* | 17 | KJ622451 |  | KJ623136 | KJ622663-64 |  |  | KJ622524 | KJ622589 | KJ622408 | KJ622816 | KJ622892 |  |  | **JN082083** | **JN082027** | **JN082161** |
| WYS089 | *him* | 17 |  |  |  |  |  |  |  |  |  |  |  |  | KJ623007-08 | **JN082084** | **JN082032** | **JN082162** |
| WYS095 | *him* | 17 |  |  |  |  |  |  |  |  |  |  |  |  |  |  | **JN082028** |  |
| WYS096 | *him* | 17 |  |  |  |  |  |  |  |  |  |  |  |  |  |  | **JN082033** | **JN082163** |
| WYS097 | *him* | 17 |  |  |  |  |  |  |  |  |  |  |  |  |  | **JN082085** | **JN082029** | **JN082164** |
| WYS098 | *him* | 17 | KJ622452 |  |  |  |  |  |  |  |  |  |  |  |  | **JN082086** | **JN082031** |  |
| WYS099 | *him* | 17 |  |  |  |  |  |  |  |  |  |  |  |  |  | **JN082087** | **JN082034** | **JN082165** |
| WYS102 | *him* | 17 |  |  |  |  |  |  |  |  |  |  |  |  |  |  | **JN082030** | **JN082166** |
| LLJ002 | *him* | 19 |  |  |  |  |  |  |  |  |  |  |  |  |  | **JN082102** | **JN082039** |  |
| LLJ012 | *him* | 19 |  |  |  |  |  |  |  |  |  |  |  |  |  | **JN082103** | **JN082040** | **JN082134** |
| LLJ034 | *him* | 19 |  |  |  |  | KJ622724 | KJ622780 | KJ622550-51 | KJ622615-16 | KJ622403 |  |  |  | KJ623019-20 | **JN082104** | **JN082041** | **JN082135** |
| LLJ035 | *him* | 19 |  |  |  |  |  |  |  |  |  |  |  |  |  |  | **JN082042** | **JN082136** |
| LLJ051 | *him* | 19 | KJ622462 |  |  |  |  |  |  |  |  |  |  |  |  | **JN082106** | **JN082043** |  |
| LLJ052 | *him* | 19 | KJ622463 |  |  |  |  |  |  |  |  |  |  |  |  | **JN082107** | **JN082044** | **JN082138** |
| LLJ055 | *him* | 19 |  |  |  |  |  |  |  |  |  |  |  |  |  | **JN082108** | **JN082045** | **JN082139** |
| LLJ081 | *him* | 19 |  |  |  |  |  |  |  |  |  |  |  |  |  | **JN082109** | **JN082046** | **JN082140** |
| LLJ050 | *him* | 19 | KJ622461 |  |  |  |  |  | KJ622517-18 | KJ622582-83 |  |  |  |  |  | **JN082105** |  | **JN082137** |
| LLJ076 | *him* | 19 |  |  | KJ623130 |  |  |  |  |  | KJ622394 |  |  | KJ622936-37 |  |  |  |  |
| LLJ077 | *him* | 19 |  |  | KJ623131-32 |  |  |  |  |  | KJ622400 |  |  |  |  |  |  |  |
| LLJ084 | *him* | 19 |  |  |  | KJ622657-58 |  |  | KJ622521-22 |  |  | KJ622822-23 |  |  |  |  |  |  |
| LLJ088 | *him* | 19 |  |  | KJ623133 | KJ622652 |  |  | KJ622519-20 | KJ622584-85 | KJ622402 | KJ622824-25 |  | KJ622938-39 |  |  |  |  |
| JLH002 | *him* | 20 | KJ622456 | KJ623074-75 | KJ623142 |  | KJ622737-38 | KJ622771 |  |  | KJ622404 | KJ622826-27 | KJ622891 | KJ622927-28 | KJ623013-14 | **JN082092** |  | **JN082141** |
| JLH004 | *him* | 20 | KJ622457 | KJ623072-73 |  | KJ622653-54 |  | KJ622772 | KJ622548-49 | KJ622613-14 | KJ622406 | KJ622828-29 |  | KJ622963 |  | **JN082093** |  | **JN082142** |
| JLH005 | *him* | 20 |  |  |  |  |  |  |  |  |  |  |  |  |  | **JN082094** |  | **JN082143** |
| JLH006 | *him* | 20 |  |  |  |  |  |  |  |  |  |  |  |  |  | **JN082095** |  | **JN082144** |
| JLH008 | *him* | 20 | KJ622458 |  |  |  |  |  |  |  |  |  |  |  |  | **JN082096** |  | **JN082145** |
| JLH013 | *him* | 20 |  |  |  |  | KJ622741-42 | KJ622790 |  |  |  |  |  |  |  |  |  |  |
| JLH014 | *him* | 20 |  |  | KJ623134-35 | KJ622655-56 |  |  | KJ622516 | KJ622581 | KJ622401 | KJ622830-31 |  |  | KJ623009-10 | **JN082097** | **JN082047** |  |
| JLH018 | *him* | 20 | KJ622459 |  |  |  |  |  |  |  |  |  |  |  |  | **JN082098** | **JN082048** | **JN082146** |
| JLH020 | *him* | 20 |  |  |  |  |  |  |  |  |  |  |  |  |  | **JN082099** | **JN082049** | **JN082147** |
| JLH021 | *him* | 20 |  |  |  |  |  |  |  |  |  |  |  |  |  | **JN082100** | **JN082050** | **JN082148** |
| JLH022 | *him* | 20 |  |  |  |  |  |  |  |  |  |  |  |  |  |  | **JN082051** |  |
| JLH023 | *him* | 20 |  |  |  |  |  |  |  |  |  |  |  |  |  |  | **JN082052** |  |
| JLH028 | *him* | 20 |  |  |  |  |  |  |  |  |  |  |  |  |  | **JN082101** | **JN082053** | **JN082149** |
| JLH019 | *him* | 20 | KJ622460 |  |  |  |  |  |  |  |  |  |  |  |  |  |  |  |
| ZY14 | *him* | 25 | KJ622443 |  | KJ623128-29 | KJ622649-50 |  |  | KJ622544-45 | KJ622609-10 | KJ622393 | KJ622846-47 |  | KJ622959-60 |  | **JN082119-20** | **JN082014** | **JN082127** |
| ZY15 | *him* | 25 | KJ622445 |  |  | KJ622647-48 | KJ622728 | KJ622783 | KJ622537 | KJ622602 | KJ622398 | KJ622844-45 |  | KJ622954 | KJ623005-06 | **JN082121-22** | **JN082015** | **JN082128** |
| ZY16 | *him* | 25 | KJ622446 |  |  |  |  |  |  |  |  |  |  |  |  | **JN082123** | **JN082016** | **JN082130** |
| ZY18 | *him* | 25 |  |  |  |  |  |  |  |  |  |  |  |  |  |  | **JN082017** |  |
| ZY26 | *him* | 25 |  |  |  |  |  |  |  |  |  |  |  |  |  |  |  | **JN082126** |
| ZY28 | *him* | 25 |  |  |  |  |  |  |  |  |  |  |  |  |  | **JN082124** |  |  |
| WLB033 | *mac* | 9 |  | KJ623096 | KJ623159-60 | KJ622697 | KJ622751 | KJ622798 | KJ622554-55 | KJ622619-20 | KJ622425 | KJ622856 | KJ622912 | KJ622967-68 | KJ623024-25 |  |  | **JN082171** |
| WLB032 | *mac* | 9 | KJ622488 |  |  |  |  |  |  |  |  |  |  |  |  |  |  | **JN082170** |
| FQX009 | *mac* | 14 | KJ622482 |  |  | KJ622707 |  |  | KJ622561 | KJ622626 | KJ622430 | KJ622852-53 |  | KJ622966 |  |  | **JN082012** |  |
| FQX010 | *mac* | 14 | KJ622483 |  |  |  |  |  |  |  |  |  |  |  |  | **JN082074** |  | **JN082169** |
| FQX011 | *mac* | 14 |  |  |  | KJ622710 |  |  |  |  |  |  |  |  |  | **JN082075** | **JN082013** | **JN082168** |
| FQX012 | *mac* | 14 |  | KJ623100 | KJ623153-54 | KJ622708-09 | KJ622752 | KJ622796 | KJ622562 | KJ622627 | KJ622424 | KJ622851 | KJ622901-02 |  | KJ623041-42 |  |  |  |
| FQX015 | *mac* | 14 |  |  |  |  | KJ622753-54 | KJ622797 |  |  |  |  | KJ622910-11 |  | KJ623027-28 |  |  |  |
| FJK004 | *mac* | 12 | KJ622478 | KJ623097 | KJ623161-62 | KJ622689 | KJ622755 | KJ622791 | KJ622557-58 | KJ622622-23 | KJ622420 | KJ622848-49 |  | KJ622975-76 | KJ623029-30 | **JN082071** | **JN082006** |  |
| FJK008 | *mac* | 12 |  |  |  |  | KJ622756-57 |  |  |  |  |  |  |  | KJ623033-34 |  | **JN082007** |  |
| FJK009 | *mac* | 12 | KJ622479 |  |  |  |  |  |  |  |  |  |  |  |  |  | **JN082008** |  |
| FJK010 | *mac* | 12 |  | KJ623098 | KJ623156-57 | KJ622690-91 |  |  |  |  | KJ622429 | KJ622855 | KJ622903 | KJ622977 |  | **JN082072** | **JN082009** |  |
| FJK014 | *mac* | 12 | KJ622480 | KJ623099 | KJ623158 | KJ622692 | KJ622758 | KJ622792 | KJ622563 | KJ622628 | KJ622421 | KJ622854 |  | KJ622978-79 | KJ623026 | **JN082073** | **JN082010** | **JN082167** |
| FJK015 | *mac* | 12 |  |  |  |  |  |  |  |  |  |  |  |  |  |  | **JN082011** |  |
| FJK020 | *mac* | 12 | KJ622481 |  |  |  |  |  |  |  |  |  |  |  |  |  |  |  |
| SLD001 | *mac* | 8 | KJ622486 |  |  |  |  |  |  |  |  |  |  |  |  |  |  |  |
| SLD003 | *mac* | 8 |  |  |  |  |  |  |  |  |  |  |  |  |  |  | **JN081998** |  |
| SLD004 | *mac* | 8 |  |  |  |  |  |  |  |  |  |  |  |  |  | **JN082066** | **JN081999** | **JN082172** |
| SLD005 | *mac* | 8 |  | KJ623103 |  |  |  | KJ622794 |  |  |  |  |  |  |  | **JN082067** |  |  |
| SLD006 | *mac* | 8 | KJ622487 |  |  |  |  |  |  |  |  |  |  |  |  |  | **JN082000** |  |
| SLD010 | *mac* | 8 |  | KJ623104 |  |  |  | KJ622795 |  |  |  |  |  |  |  | **JN082068** | **JN082001** | **JN082174** |
| SLD012 | *mac* | 8 |  |  |  | KJ622696 |  |  |  |  |  |  |  |  |  | **JN082069-70** |  | **JN082173** |
| SLD017 | *mac* | 8 |  |  |  |  | KJ622748 |  |  |  |  |  |  |  | KJ623037-38 |  |  |  |
| SLD019 | *mac* | 8 |  |  |  | KJ622693-94 |  |  | KJ622567 | KJ622632 | KJ622423 | KJ622859-60 | KJ622904-05 | KJ622971-72 |  |  |  |  |
| SLD020 | *mac* | 8 |  |  | KJ623163-64 | KJ622695 |  |  | KJ622568 | KJ622633 | KJ622422 | KJ622857-58 | KJ622906-07 | KJ622973-74 | KJ623035-36 |  |  |  |
| SLD021 | *mac* | 8 |  |  |  |  | KJ622749 |  |  |  |  |  |  |  | KJ623031-32 |  |  |  |
| SLD022 | *mac* | 8 |  | KJ623105 | KJ623152 |  | KJ622750 |  |  |  | KJ622432 |  |  |  | KJ623023 |  |  |  |
| B019 | *mac* | 6 | KJ622490 | KJ623095 | KJ623155 | KJ622702-03 | KJ622759-60 | KJ622800 | KJ622556 | KJ622621 | KJ622431 | KJ622850 |  |  |  |  | **JN082003-04** | **JN082176** |
| B067 | *mac* | 6 |  |  |  |  |  |  |  |  |  |  |  |  |  |  | **JN082005** |  |
| LF01 | *mac* | 10 |  |  |  |  |  |  |  |  |  |  |  |  |  | **JN082065** |  |  |
| LF007 | *mac* | 10 | KJ622484 |  | KJ623151 | KJ622700-01 | KJ622747 | KJ622793 | KJ622564-65 | KJ622629-30 | KJ622419 | KJ622861-62 | KJ622908-09 |  | KJ623039-40 |  |  | **JN082175** |
| LF020 | *mac* | 10 | KJ622485 |  |  | KJ622698-99 |  |  | KJ622566 | KJ622631 | KJ622428 | KJ622863-64 |  | KJ622969-70 |  |  |  |  |
| NBCP015 | *mac* | 7 |  | KJ623102 | KJ623165 | KJ622705-06 |  | KJ622799 | KJ622560 | KJ622625 | KJ622427 | KJ622865-66 | KJ622898-99 |  |  | **JN082064** |  |  |
| NBCP001 | *mac* | 7 | KJ622489 | KJ623101 |  | KJ622704 |  |  | KJ622559 | KJ622624 | KJ622426 |  | KJ622900 | KJ622964-65 |  | **JN082063** | **JN082002** | **JN082177** |
| DL163 | *hai* | 35 |  | KJ623118 |  |  |  |  |  |  |  |  |  |  |  |  |  |  |
| DL598 | *hai* | 28 | KJ622495 |  |  |  |  |  |  |  |  |  |  |  |  |  | KJ622390 |  |
| DL601 | *hai* | 28 |  | KJ623106-07 |  |  |  |  |  |  |  |  |  |  |  |  |  |  |
| DL602 | *hai* | 28 |  |  |  |  |  |  |  |  |  |  |  |  |  |  | KJ622372-73 |  |
| DL605 | *hai* | 28 | KJ622496 |  |  |  |  |  |  |  |  |  |  |  |  |  |  |  |
| DL606 | *hai* | 28 |  |  |  |  |  |  |  |  |  |  |  |  |  |  | KJ622374 |  |
| DL610 | *hai* | 28 |  |  |  |  |  |  |  |  |  |  |  |  |  |  | KJ622375 |  |
| DL615 | *hai* | 28 |  |  | KJ623170-71 | KJ622714 | KJ622761 | KJ622806 | KJ622575-76 | KJ622640-41 | KJ622435 | KJ622876 | KJ622919-20 | KJ622983-84 | KJ623051-52 | KJ623059 |  | KJ623185 |
| JC390 | *hai* | 31 |  | KJ623110 | KJ623179-80 | KJ622715 |  | KJ622804 | KJ622577 | KJ622642 | KJ622436 |  | KJ622918 | KJ622980 | KJ623058 |  |  | KJ623191 |
| JC396 | *hai* | 31 | KJ622497 |  |  |  |  |  |  |  |  |  |  |  |  |  |  |  |
| JC401 | *hai* | 31 | KJ622498 |  |  |  |  |  |  |  |  |  |  |  |  |  | KJ622376 |  |
| JC403 | *hai* | 31 |  |  |  |  |  |  |  |  |  |  |  |  |  |  | KJ622377 |  |
| JC405 | *hai* | 31 | KJ622499 |  |  |  |  |  |  |  |  |  |  |  |  |  |  |  |
| JC406 | *hai* | 31 | KJ622500 |  |  |  |  |  |  |  |  |  |  |  |  |  |  |  |
| XM197 | *hai* | 32 |  | KJ623120 |  |  |  |  |  |  |  |  |  |  |  |  |  |  |
| XM198 | *hai* | 32 |  | KJ623112-13 | KJ623177-78 | KJ622716-17 | KJ622769 | KJ622803 | KJ622578 | KJ622643 | KJ622437 | KJ622877 | KJ622925-26 | KJ622989-90 | KJ623043-44 | KJ623064 |  |  |
| XM208 | *hai* | 32 | KJ622512 |  |  |  |  |  |  |  |  |  |  |  |  |  |  |  |
| XM214 | *hai* | 32 | KJ622513 |  |  |  |  |  |  |  |  |  |  |  |  |  |  |  |
| QE002 | *hai* | 26 |  |  |  |  |  |  |  |  |  |  |  |  |  |  | KJ622384-85 |  |
| QE003 | *hai* | 26 |  | KJ623126 | KJ623175 | KJ622719 | KJ622764 | KJ622807 | KJ622569-70 | KJ622634-35 | KJ622439 | KJ622869 | KJ622917 | KJ622987-88 | KJ623056-57 | KJ623060 |  | KJ623188 |
| QE004 | *hai* | 26 | KJ622491 |  |  |  |  |  |  |  |  |  |  |  |  | KJ623061 | KJ622386 | KJ623184 |
| QE005 | *hai* | 26 | KJ622492 |  |  |  |  |  |  |  |  |  |  |  |  |  |  |  |
| QE006 | *hai* | 26 | KJ622493 |  |  |  |  |  |  |  |  |  |  |  |  |  | KJ622387 |  |
| XL002 | *hai* | 36 |  |  |  |  |  |  |  |  |  |  |  |  |  |  | KJ622391 |  |
| XL005 | *hai* | 36 |  | KJ623124-25 | KJ623168-69 | KJ622723 | KJ622768 |  | KJ622573 | KJ622638 | KJ622434 | KJ622874-75 | KJ622921-22 | KJ622997-98 | KJ623047-48 |  |  |  |
| XL010 | *hai* | 36 | KJ622494 |  |  |  |  |  |  |  |  |  |  |  |  |  |  |  |
| XL014 | *hai* | 36 |  | KJ623117 |  |  |  |  |  |  |  |  |  |  |  |  |  |  |
| XL015 | *hai* | 36 |  | KJ623119 |  |  |  |  |  |  |  |  |  |  |  |  |  |  |
| SL20 | *hai* | 34 | KJ622509 | KJ623123 | KJ623173-74 | KJ622720-21 | KJ622766-67 | KJ622808 | KJ622574 | KJ622639 |  | KJ622872-73 | KJ622923-24 |  | KJ623045-46 | KJ623063 |  |  |
| SL27 | *hai* | 34 | KJ622510 |  |  |  |  |  |  |  |  |  |  |  |  |  |  |  |
| SL28 | *hai* | 34 | KJ622511 |  |  |  |  |  |  |  |  |  |  |  |  |  |  |  |
| LZ539 | *hai* | 27 |  | KJ623109 |  |  |  |  |  |  |  |  |  |  |  |  |  |  |
| LZ540 | *hai* | 27 |  | KJ623116 | KJ623172 | KJ622718 | KJ622765 | KJ622801 | KJ622579-80 | KJ622644-45 | KJ622438 | KJ622878-79 |  | KJ622985-86 | KJ623054-55 |  |  |  |
| LZ541 | *hai* | 27 |  |  |  |  |  |  |  |  |  |  |  |  |  |  |  | KJ623183 |
| LZ543 | *hai* | 27 | KJ622501 |  |  |  |  |  |  |  |  |  |  |  |  |  |  |  |
| LZ549 | *hai* | 27 | KJ622504 |  |  |  |  |  |  |  |  |  |  |  |  |  |  |  |
| LZ545 | *hai* | 27 | KJ622502 |  |  |  |  |  |  |  |  |  |  |  |  |  | KJ622378 |  |
| LZ547 | *hai* | 27 | KJ622503 |  |  |  |  |  |  |  |  |  |  |  |  |  | KJ622379 |  |
| LZ548 | *hai* | 27 |  |  |  |  |  |  |  |  |  |  |  |  |  |  | KJ622380 |  |
| MC186 | *hai* | 33 |  |  |  |  |  |  |  |  |  |  |  |  |  |  | KJ622381-82 |  |
| MC187 | *hai* | 33 | KJ622505 | KJ623114-15 |  |  |  |  |  |  |  |  |  |  |  |  |  |  |
| MC192 | *hai* | 33 |  |  |  |  |  |  |  |  |  |  |  |  |  |  | KJ622383 |  |
| YG006 | *hai* | 29 |  |  |  | KJ622711 |  |  | KJ622571 | KJ622636 | KJ622440 | KJ622871 |  | KJ622991-92 |  |  |  |  |
| YG007 | *hai* | 29 |  |  | KJ623176 |  |  |  |  |  | KJ622442 |  |  | KJ622993-94 |  |  |  |  |
| YG08 | *hai* | 29 |  |  | KJ623166-67 |  |  |  |  |  | KJ622441 |  |  |  |  |  |  |  |
| YG010 | *hai* | 29 |  |  |  |  |  |  |  |  |  |  |  |  |  |  | KJ622392 |  |
| YG018 | *hai* | 29 |  | KJ623121 |  | KJ622712-13 | KJ622770 |  |  |  |  | KJ622870 |  | KJ622995-96 | KJ623053 |  |  |  |
| YG13 | *hai* | 29 |  |  |  |  |  | KJ622805 |  |  |  |  |  |  |  |  |  |  |
| YG14 | *hai* | 29 |  |  |  |  |  |  |  |  |  |  |  |  |  |  |  | KJ623187 |
| YG22 | *hai* | 29 |  |  |  |  |  |  |  |  |  |  | KJ622915-16 |  |  | KJ623065 |  | KJ623186 |
| YGL433 | *hai* | 29 | KJ622514 |  |  |  |  |  |  |  |  |  |  |  |  |  |  | KJ623190 |
| YGL434 | *hai* | 29 | KJ622515 |  |  |  |  |  |  |  |  |  |  |  |  |  |  |  |
| SK262 | *hai* | 30 |  | KJ623108 |  |  |  |  |  |  |  |  |  |  |  |  |  |  |
| SK363 | *hai* | 30 | KJ622506 | KJ623111 |  |  |  |  |  |  |  |  |  |  |  |  |  |  |
| SK364 | *hai* | 30 | KJ622507 |  |  |  |  |  |  |  |  |  |  |  |  |  |  |  |
| SK366 | *hai* | 30 | KJ622508 | KJ623122 | KJ623181-82 | KJ622722 |  |  | KJ622572 | KJ622637 | KJ622433 | KJ622867-68 | KJ622913-14 | KJ622981-82 |  |  | KJ622388-89 |  |
| SK365 | *hai* | 30 |  |  |  |  | KJ622762-63 | KJ622802 |  |  |  |  |  |  | KJ623049-50 | KJ623062 |  | KJ623189 |
