# Supplementary material for: Differential introgression among loci across a hybrid zone of the intermediate horseshoe bat (Rhinolophus affinis)
Source: BMC Evol Biol. 2014 Jul 9;14:154. doi: 10.1186/1471-2148-14-154 (PMC4105523; doi:10.1186/1471-2148-14-154)
Supplement: Additional file 4: Table S4 — Parameters used for nuclear markers in coalescent simulations. [file 1471-2148-14-154-S4.doc]

Additional files:

Additional file 4: Detailed information of the forearm and echolocation call frequency for each individual used in this study.

| No. | Taxon | Individual ID | Locality number | Sex | Forearm  (mm) | Call frequency  (kHz) |
| --- | --- | --- | --- | --- | --- | --- |
| 1 | *R. a. himalayanus* | XGD10 | 3 | Male | 51.84 | 83.2 |
| 2 | *R. a. himalayanus* | XGD14 | 3 | Male | 50.2 | 82.9 |
| 3 | *R. a. himalayanus* | XGD15 | 3 | Male | 54.34 | 82.6 |
| 4 | *R. a. himalayanus* | XGD01 | 3 | Male | 52.82 | 82.8 |
| 5 | *R. a. himalayanus* | XGD05 | 3 | Female | 54.54 | 83.2 |
| 6 | *R. a. himalayanus* | XGD06 | 3 | Female | 52.92 | 83.7 |
| 7 | *R. a. himalayanus* | XGD09 | 3 | Female | 51.66 | 83.2 |
| 8 | *R. a. himalayanus* | FJK005 | 12 | Male | 51.98 | 86.1 |
| 9 | *R. a. himalayanus* | FJK022 | 12 | Female | 53.31 | 85.9 |
| 10 | *R. a. himalayanus* | FGS002 | 13 | Female | 52.98 | 86.1 |
| 11 | *R. a. himalayanus* | YH04 | 15 | Female | 52.29 | 86 |
| 12 | *R. a. himalayanus* | FWY009 | 16 | Female | 52.63 | 83.3 |
| 13 | *R. a. himalayanus* | FWY010 | 16 | Female | 55.9 | 85.9 |
| 14 | *R. a. himalayanus* | FSC01 | 18 | Male | 52.51 | 87.9 |
| 15 | *R. a. himalayanus* | FSC02 | 18 | Male | 51.11 | 87.6 |
| 16 | *R. a. himalayanus* | FSC03 | 18 | Male | 52.8 | 87.9 |
| 17 | *R. a. himalayanus* | FSC06 | 18 | Male | 52.85 | 87.6 |
| 18 | *R. a. himalayanus* | FSC11 | 18 | Male | 53 | 87.6 |
| 19 | *R. a. himalayanus* | FSC12 | 18 | Male | 50.08 | 87.6 |
| 20 | *R. a. himalayanus* | FSC13 | 18 | Male | 51.22 | 87.6 |
| 21 | *R. a. himalayanus* | LLJ073 | 19 | Male | 51.722 | 88.8 |
| 22 | *R. a. himalayanus* | LLJ079 | 19 | Female | 51.62 | 88.8 |
| 23 | *R. a. himalayanus* | LLJ080 | 19 | Male | 50.19 | 88.8 |
| 24 | *R. a. himalayanus* | JLH002 | 20 | Male | 51.5 | 88.3 |
| 25 | *R. a. himalayanus* | JLH008 | 20 | Male | 52 | 88.3 |
| 26 | *R. a. himalayanus* | JLH013 | 20 | Male | 50.3 | 88.1 |
| 27 | *R. a. himalayanus* | JLH019 | 20 | Male | 52.4 | 88.1 |
| 28 | *R. a. himalayanus* | JLH021 | 20 | Male | 52 | 88.1 |
| 29 | *R. a. himalayanus* | JLH001 | 20 | Female | 51 | 88.3 |
| 30 | *R. a. himalayanus* | JLH004 | 20 | Female | 50.5 | 88.1 |
| 31 | *R. a. himalayanus* | JLH005 | 20 | Female | 52 | 88.5 |
| 32 | *R. a. himalayanus* | JLH006 | 20 | Female | 51 | 88.1 |
| 33 | *R. a. himalayanus* | JLH018 | 20 | Female | 51 | 88.3 |
| 34 | *R. a. himalayanus* | JLH020 | 20 | Female | 51.8 | 88.1 |
| 35 | *R. a. himalayanus* | BSC01 | 21 | Male | 53.2 | 86.8 |
| 36 | *R. a. himalayanus* | YL24 | 24 | Male | 54.26 | 88.8 |
| 37 | *R. a. himalayanus* | ZY23 | 25 | Male | 52.79 | 88.8 |
| 38 | *R. a. himalayanus* | ZY21 | 25 | Female | 52.08 | 89 |
| 39 | *R. a. himalayanus* | ZY22 | 25 | Female | 54.31 | 88.8 |
| 40 | *R. a. himalayanus* | ZY25 | 25 | Female | 50.17 | 88.8 |
| 41 | *R. a. himalayanus* | ZY26 | 25 | Female | 52.43 | 88.8 |
| 42 | *R. a. himalayanus* | ZY28 | 25 | Female | 52.22 | 88.8 |
| 43 | *R. a. himalayanus* | ZY31 | 25 | Female | 52.06 | 88.8 |
| 1 | *R. a. macrurus* | SLD07006 | 8 | Male | 52 | 74.1 |
| 2 | *R. a. macrurus* | SLD07009 | 8 | Male | 52 | 73.9 |
| 3 | *R. a. macrurus* | SLD07012 | 8 | Male | 54.7 | 74.2 |
| 4 | *R. a. macrurus* | SLD07014 | 8 | Male | 53.9 | 73.8 |
| 5 | *R. a. macrurus* | SLD07015 | 8 | Male | 54.1 | 74.2 |
| 6 | *R. a. macrurus* | SLD07018 | 8 | Male | 52.1 | 73.6 |
| 7 | *R. a. macrurus* | SLD07019 | 8 | Male | 52.6 | 73.6 |
| 8 | *R. a. macrurus* | SLD07024 | 8 | Male | 53.2 | 73.8 |
| 9 | *R. a. macrurus* | SLD07025 | 8 | Male | 52 | 73.8 |
| 10 | *R. a. macrurus* | SLD07033 | 8 | Male | 53.9 | 74.3 |
| 11 | *R. a. macrurus* | SLD07039 | 8 | Male | 50.2 | 73.6 |
| 12 | *R. a. macrurus* | SLD07001 | 8 | Female | 53 | 74.2 |
| 13 | *R. a. macrurus* | SLD07002 | 8 | Female | 50.6 | 74.3 |
| 14 | *R. a. macrurus* | SLD07003 | 8 | Female | 52.8 | 74.3 |
| 15 | *R. a. macrurus* | SLD07004 | 8 | Female | 51.4 | 74.2 |
| 16 | *R. a. macrurus* | SLD07005 | 8 | Female | 52.4 | 74.1 |
| 17 | *R. a. macrurus* | SLD07010 | 8 | Female | 53.4 | 74.2 |
| 18 | *R. a. macrurus* | SLD07011 | 8 | Female | 52.5 | 74.2 |
| 19 | *R. a. macrurus* | SLD07016 | 8 | Female | 52.1 | 74.2 |
| 20 | *R. a. macrurus* | SLD07017 | 8 | Female | 51.2 | 74.3 |
| 21 | *R. a. macrurus* | SLD07020 | 8 | Female | 52 | 74.1 |
| 22 | *R. a. macrurus* | SLD07021 | 8 | Female | 51.7 | 74.2 |
| 23 | *R. a. macrurus* | SLD07022 | 8 | Female | 52.1 | 74.2 |
| 24 | *R. a. macrurus* | SLD07023 | 8 | Female | 51.3 | 74.1 |
| 25 | *R. a. macrurus* | SLD07031 | 8 | Female | 50 | 74.3 |
| 26 | *R. a. macrurus* | SLD07032 | 8 | Female | 51 | 74.1 |
| 27 | *R. a. macrurus* | SLD07034 | 8 | Female | 50 | 74.3 |
| 28 | *R. a. macrurus* | SLD07043 | 8 | Female | 51.2 | 74.3 |
| 29 | *R. a. macrurus* | LF007 | 10 | Male | 51.7 | 73.4 |
| 30 | *R. a. macrurus* | LF020 | 10 | Female | 49.2 | 73.6 |
| 31 | *R. a. macrurus* | FKD003 | 11 | Female | 52.1 | 72.2 |
| 32 | *R. a. macrurus* | FJK008 | 12 | Male | 52.29 | 72.2 |
| 33 | *R. a. macrurus* | FJK010 | 12 | Male | 52.52 | 72.4 |
| 34 | *R. a. macrurus* | FJK012 | 12 | Male | 53.68 | 72.2 |
| 35 | *R. a. macrurus* | FJK004 | 12 | Female | 52.57 | 72.7 |
| 36 | *R. a. macrurus* | FJK009 | 12 | Female | 54.81 | 72.5 |
| 37 | *R. a. macrurus* | FJK015 | 12 | Female | 53.13 | 71.9 |
| 38 | *R. a. macrurus* | FQX009 | 14 | Male | 53.95 | 72.8 |
| 39 | *R. a. macrurus* | FQX010 | 14 | Female | 52.32 | 73.2 |
| 1 | *R. a. hainanus* | LZ540 | 27 | Female | 51.6 | 72.9 |
| 2 | *R. a. hainanus* | DL591 | 28 | Female | 50.9 | 72.9 |
| 3 | *R. a. hainanus* | YG006 | 29 | Male | 49.9 | 71 |
| 4 | *R. a. hainanus* | YG009 | 29 | Male | 52.9 | 71 |
| 5 | *R. a. hainanus* | YG010 | 29 | Male | 51.3 | 71 |
| 6 | *R. a. hainanus* | YG011 | 29 | Male | 50.2 | 71 |
| 7 | *R. a. hainanus* | YG012 | 29 | Male | 50.3 | 71 |
| 8 | *R. a. hainanus* | YG001 | 29 | Female | 51.6 | 71 |
| 9 | *R. a. hainanus* | YG004 | 29 | Female | 52.6 | 71 |
| 10 | *R. a. hainanus* | YG005 | 29 | Female | 49.6 | 71 |
| 11 | *R. a. hainanus* | YG007 | 29 | Female | ? | 71 |
| 12 | *R. a. hainanus* | YG008 | 29 | Female | ? | 71 |
| 13 | *R. a. hainanus* | SK362 | 30 | Female | 52.4 | 72.9 |
| 14 | *R. a. hainanus* | SK363 | 30 | Male | 50.8 | 72.9 |
| 15 | *R. a. hainanus* | JC391 | 31 | Female | 48.8 | 70.9 |
| 16 | *R. a. hainanus* | HL002 | 31 | Female | 49.7 | 71 |
| 17 | *R. a. hainanus* | XMSK197 | 32 | Female | 51.9 | 70.9 |
| 18 | *R. a. hainanus* | NX001 | 35 | Female | 51.8 | 71 |
| 19 | *R. a. hainanus* | NX129 | 35 | Male | 52.3 | 70 |
| 20 | *R. a. hainanus* | DL163 | 35 | Male | 51.1 | 71.8 |
| 21 | *R. a. hainanus* | XL003 | 36 | Male | 49.8 | 70 |
| 22 | *R. a. hainanus* | XL007 | 36 | Male | 50.4 | 70 |
| 23 | *R. a. hainanus* | XL010 | 36 | Male | 51.4 | 70 |
| 24 | *R. a. hainanus* | XL011 | 36 | Male | 52.4 | 70 |
| 25 | *R. a. hainanus* | XL012 | 36 | Male | 51.3 | 70 |
| 26 | *R. a. hainanus* | XL014 | 36 | Male | 51.3 | 70 |
| 27 | *R. a. hainanus* | XL015 | 36 | Male | 50 | 70 |
| 28 | *R. a. hainanus* | XL002 | 36 | Female | 52.8 | 70 |
| 29 | *R. a. hainanus* | XL004 | 36 | Female | 51.3 | 70 |
| 30 | *R. a. hainanus* | XL005 | 36 | Female | 51.7 | 70 |
| 31 | *R. a. hainanus* | XL006 | 36 | Female | 51.2 | 70 |
| 32 | *R. a. hainanus* | XL009 | 36 | Female | 50.1 | 70 |
