# Supplementary material for: Differential introgression among loci across a hybrid zone of the intermediate horseshoe bat (Rhinolophus affinis)
Source: BMC Evol Biol. 2014 Jul 9;14:154. doi: 10.1186/1471-2148-14-154 (PMC4105523; doi:10.1186/1471-2148-14-154)
Supplement: Additional file 5: Table S5 — Tests of nested models for migration rates between himalayanus and macrurus based on the full data set. [file 1471-2148-14-154-S5.doc]

Additional files:

Additional file 5: Tests of nested models for migration rates between *himalayanus* and *macrurus* based on the full data set.

| Locus | Model | d.f. | 2LLR* | P |
| --- | --- | --- | --- | --- |
| *Prestin-4* | m_hm =_ m_mh_ | 1 | -1.243 | 0.265 |
|  | m_hm = 0_ | 1 | 0.001 | 1.000 |
|  | m_mh = 0_ | 1 | 4.076 | 0.043 |
|  | m_hm =_ m_mh = 0_ | 2 | 5.333 | 0.069 |
| *Prestin-8* | m_hm =_ m_mh_ | 1 | 2.537 | 0.135 |
|  | m_hm = 0_ | 1 | 3.221 | 0.073 |
|  | m_mh = 0_ | 1 | 0.001 | 1.000 |
|  | m_hm =_ m_mh = 0_ | 2 | 4.102 | 0.129 |
| *Prestin-18* | m_hm =_ m_mh_ | 1 | 1.623 | 0.203 |
|  | m_hm = 0_ | 1 | 0.1374 | 0.711 |
|  | m_mh = 0_ | 1 | 0.4336 | 0.510 |
|  | m_hm =_ m_mh = 0_ | 2 | 3.093 | 0.213 |
| *Pola1* | m_hm =_ m_mh_ | 1 | -0.6363 | 0.425 |
|  | m_hm = 0_ | 1 | 0.001 | 1.000 |
|  | m_mh = 0_ | 1 | 3.333 | 0.067 |
|  | m_hm =_ m_mh = 0_ | 2 | 4.920 | 0.085 |
| *H2a* | m_hm =_ m_mh_ | 1 | 0.001 | 1.000 |
|  | m_hm = 0_ | 1 | 0.001 | 1.000 |
|  | m_mh = 0_ | 1 | 0.001 | 1.000 |
|  | m_hm =_ m_mh = 0_ | 2 | 0.001 | 1.000 |
| *Sws1* | m_hm =_ m_mh_ | 1 | 1.593 | 0.207 |
|  | m_hm = 0_ | 1 | 1.593 | 0.207 |
|  | m_mh = 0_ | 1 | 0.001 | 1.000 |
|  | m_hm =_ m_mh = 0_ | 2 | 1.593 | 0.451 |
| *Thy* | m_hm =_ m_mh_ | 1 | 0.8393 | 0.360 |
|  | m_hm = 0_ | 1 | 3.635 | 0.056 |
|  | m_mh = 0_ | 1 | 0.001 | 1.000 |
|  | m_hm =_ m_mh = 0_ | 2 | 11.211 | 0.004 |
| *Tg* | m_hm =_ m_mh_ | 1 | 2.047 | 0.153 |
|  | m_hm = 0_ | 1 | 2.292 | 0.130 |
|  | m_mh = 0_ | 1 | 3.597 | 0.058 |
|  | m_hm =_ m_mh = 0_ | 2 | 5.610 | 0.061 |
| *Kcnq4* | m_hm =_ m_mh_ | 1 | 0.3026 | 0.582 |
|  | m_hm = 0_ | 1 | 0.3165 | 0.574 |
|  | m_mh = 0_ | 1 | 0.001 | 1.000 |
|  | m_hm =_ m_mh = 0_ | 2 | 1.648 | 0.439 |

m_hm_ means migration rates from *himalayanus* to *macrurus*; m_mh_ means migration rates from *macrurus* to *himalayanus*. * The log likelihood ratio (LLR) statistics of the nested model was calculated in IMa2 and associated P values were estimated from a chi-squared distribution of 2LLR with the degree of freedom (d.f.).
